# Insulin-Like Factor 3 and the HPG Axis in the Male

**DOI:** 10.3389/fendo.2014.00006

**Published:** 2014-01-27

**Authors:** Richard Ivell, Kee Heng, Ravinder Anand-Ivell

**Affiliations:** ^1^School of Molecular and Biomedical Science, University of Adelaide, Adelaide, SA, Australia; ^2^Leibniz Institute for Farm Animal Biology, Dummerstorf, Germany; ^3^School of Biosciences, University of Nottingham, Nottingham, UK

**Keywords:** INSL3, RXFP2, Leydig cell, testosterone, puberty, hypothalamic hypogonadism

## Abstract

The hypothalamic–pituitary–gonadal (HPG) axis comprises pulsatile GnRH from the hypothalamus impacting on the anterior pituitary to induce expression and release of both LH and FSH into the circulation. These in turn stimulate receptors on testicular Leydig and Sertoli cells, respectively, to promote steroidogenesis and spermatogenesis. Both Leydig and Sertoli cells exhibit negative feedback to the pituitary and/or hypothalamus via their products testosterone and inhibin B, respectively, thereby allowing tight regulation of the HPG axis. In particular, LH exerts both acute control on Leydig cells by influencing steroidogenic enzyme activity, as well as chronic control by impacting on Leydig cell differentiation and gene expression. Insulin-like peptide 3 (INSL3) represents an additional and different endpoint of the HPG axis. This Leydig cell hormone interacts with specific receptors, called RXFP2, on Leydig cells themselves to modulate steroidogenesis, and on male germ cells, probably to synergize with androgen-dependent Sertoli cell products to support spermatogenesis. Unlike testosterone, INSL3 is not acutely regulated by the HPG axis, but is a constitutive product of Leydig cells, which reflects their number and/or differentiation status and their ability therefore to produce various factors including steroids, together this is referred to as Leydig cell functional capacity. Because INSL3 is not subject to the acute episodic fluctuations inherent in the HPG axis itself, it serves as an excellent marker for Leydig cell differentiation and functional capacity, as in puberty, or in monitoring the treatment of hypogonadal patients, and at the same time buffering the HPG output.

## Introduction

**Figure 1:**
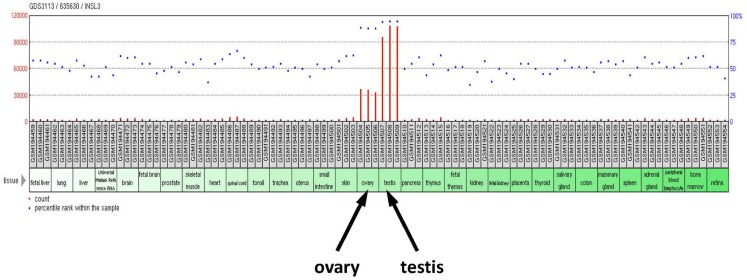
**Human tissue RNA profile based on Affymetrix microarrays (GEO profile database; GDS 3113/635630) probed for INSL3 gene expression**. Significant INSL3 mRNA is only evident for testes and ovary samples. All tissues are represented in triplicate.

Insulin-like factor 3 (INSL3) is a member of the peptide hormone family, which also includes insulin, IGF1 and IGF2, and relaxin, besides a small number of less well-known peptides (1, 2). There is insecurity about its precise structure *in vivo*. It has a very similar structure to insulin or relaxin, being made as a prepro-hormone, which after intracellular folding becomes post-translationally processed, to give rise to either an A–B heterodimeric peptide, like insulin, or possibly to an uncleaved B–C–A version, analogous to the IGFs. Why this is unclear is that both forms have been identified in the circulation of male mammals (3–5), and both forms are fully and equally bioactive (4). In the male mammal, the major site of INSL3 synthesis is the interstitial Leydig cells of both the fetal and the adult testis [Ref. (6); Figure 1]. There may be other sites of local synthesis in some peripheral tissues, but these do not contribute to the circulating levels of the hormone, which are exclusively derived from the testes, and could only have local autocrine or paracrine effects. Leydig cells are known for their production of androgenic steroids, of which testosterone (T), androstenedione (A4), and the derivative dihydrotestosterone (DHT) are the best characterized. However, besides contributing steroids to the circulation, Leydig cells also secrete large amounts of INSL3, giving rise to circulating concentrations of ca. 1 ng/ml in adult men (7–9), and higher levels in some other mammals (10, 11).

**Figure 2:**
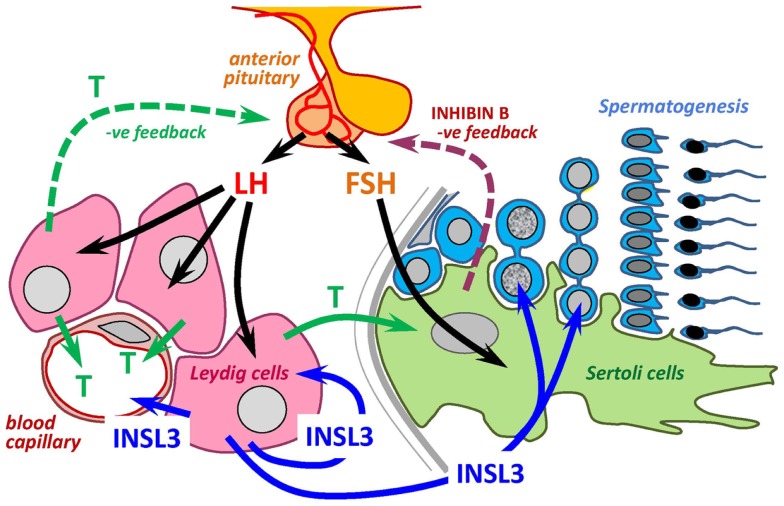
**INSL3 and HPG axis**. Scheme to show the relationship between the INSL3/RXFP2 system and testosterone as endpoint effectors of the HPG axis within the testis. Arrows are directed only to cells where there are known to be specific cognate receptors.

Thus, we need to reconsider the complexity of the hypothalamic–pituitary–gonadal (HPG) axis (Figure 2), since the gonads produce not only androgens, but also a major peptide hormone, INSL3. We still know very little about the functions attributable to INSL3, except that unlike testosterone there does not appear to be any negative feedback modulation of the hypothalamo-pituitary axis, although this has still not been very thoroughly investigated. Currently, INSL3 appears to have a systemic effect as well as both autocrine and paracrine effects within the testes themselves, in each case providing evidence for some kind of modulation of or by the classical HPG informational output, testosterone.

## INSL3 in the Male Fetus

Insulin-like factor 3 is a major product of fetal Leydig cells in all mammals so far investigated [reviewed in Ref. (6)], beginning its production shortly after sex determination and the expression of the key transcription factor SF-1 (steroidogenic factor-1). This represents about embryonic day 12 in the mouse, or week 11–12 of human pregnancy, effectively concurrent with the first detection of fetal androgens (12). In both the fetal testis as well as the adult testis, the production of INSL3 occurs only following a certain maturational differentiation of the Leydig cells. Whereas in the human fetus, as in the adults of all mammals, this differentiation appears to be dependent on the gonadotropin LH, but this is not the case for the mouse. In the fetal mouse, Leydig cell differentiation is independent of LH production, but rather appears to be regulated by the adrenocorticotropic hormone ACTH (13), even though LH receptors may be present (14). A good illustration of this is the observation that INSL3 levels in fetal Leydig cells from hypogonadal (*hpg*, *gnrh*^−/−^) mice are indistinguishable from those of wild type mice, even though LH levels are very low (15).

The main function of INSL3 in the male fetus is to induce the first, transabdominal phase of testicular descent, which ensues shortly after sex determination and concomitant with the first appearance of INSL3 or its mRNA in the fetus or in amniotic fluid (12). INSL3 acts on its unique receptor RXFP2 (relaxin family peptide receptor 2), which is a G-protein coupled receptor normally linked to G_s_, activating adenylyl cyclase (1), and which in the male fetus is expressed by the cells of the gubernacular bulb. The gubernaculum is the ligament connecting the ventral aspect of the developing testis with the inguinal region. Activation of RXFP2 causes a thickening of the gubernacular bulb, which loses elasticity, and effectively retains the once perirenal testis in the inguinal region, at a time when other somatic development is causing the kidney and neighboring organs to grow away in an antero-dorsal direction. Although an active HPG axis is not essential for this process in mice, androgens act synergistically with INSL3 to achieve this important developmental step (16). Partly, it appears that androgens are required to induce the RXFP2 receptors (17, 18), and partly it seems that both androgens and INSL3 share very similar effector signaling pathways (19). INSL3 is not required for the subsequent inguino-scrotal migration of the testis, which appears to require only androgens, or at least an active HPG axis (20).

## INSL3 at Puberty and in the Adult

Following testicular descent at or after birth, the fetal Leydig cells mostly involute. Apart from the so-called “minipuberty” in humans at about 3 months of age, when Leydig cells appear to be transiently active again (21), the testes remain steroidogenically quiescent until puberty begins. The adult population of Leydig cells represent a completely separate lineage of cells from the fetal population, though presumably may share common Leydig stem cells with these. Adult-type Leydig cells differentiate during puberty in an LH-dependent manner, dependent both on the increasing production and pulse frequency of pituitary LH, as well as on the expression of full-length functional LH receptors by the immature Leydig cells. This latter feature is important to emphasize since early Leydig cell stages, at least in rodents, appear to express large amounts of non-functional truncated LH receptor gene transcripts (22–24).

**Figure 3:**
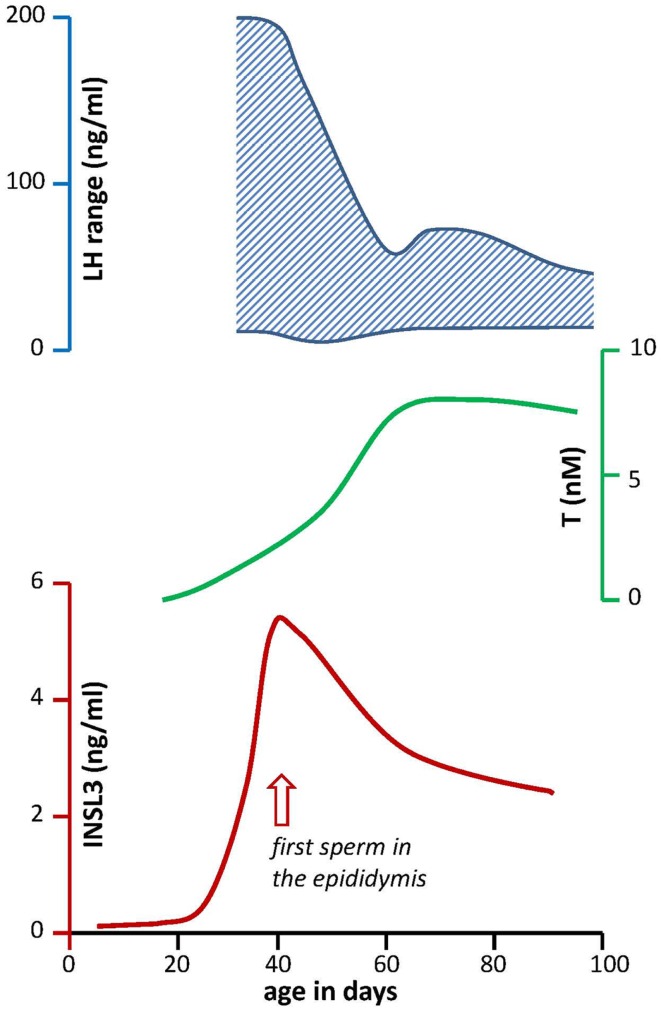
**Profiles through rat post-natal development for key circulating hormones of the HPG axis**. LH (upper panel) is given as range to indicate the high degree of episodic secretion during early puberty, which is not represented in simple mean values (26). Testosterone (T; middle panel) concentrations are derived from Bartlett et al. (27) based on simple radioimmunassay. The profile for circulating INSL3 [lower panel; Ref. (10)] indicates the marked “overshoot” during early puberty, corresponding to the high LH variance (upper panel). Note that INSL3 values reduce to a stable lower concentration, concomitant with the asymptotic testosterone maximum, and the reduction in LH episodic fluctuation.

During puberty, the HPG axis becomes hyperactivated, with large and more frequent pulses of LH causing the synthesis and secretion of large amounts of testosterone, which in turn feedback on the pituitary and hypothalamus to regulate LH pulsatility (25). In rats, this is best illustrated less by changes in mean LH values, but rather by the range of LH concentration (Figure 3), which reflects the strong episodic secretion of LH during early puberty and becomes substantially reduced as puberty progresses (26). The average circulating testosterone levels follow a simple asymptotic curve as illustrated in Figure 3. This is the resultant both of chronic LH-dependent Leydig cell differentiation, causing long-term induction of appropriate steroidogenic genes, and acute androgen-dependent feedback mechanisms regulating acute LH pulse-dependent and consequent cAMP (PKA)-dependent regulation of steroidogenic enzyme activity. This is different for what happens to INSL3 (Figure 3). INSL3 production appears to follow the anatomical differentiation of Leydig cells consequent upon the massive pubertal LH pulsatility, and peaks at around day 40 in the rat, then subsequently declines to stabilize at a lower circulating concentration as the HPG axis attains its stable adult configuration, with the maximal testosterone output and negative feedback.

**Figure 4:**
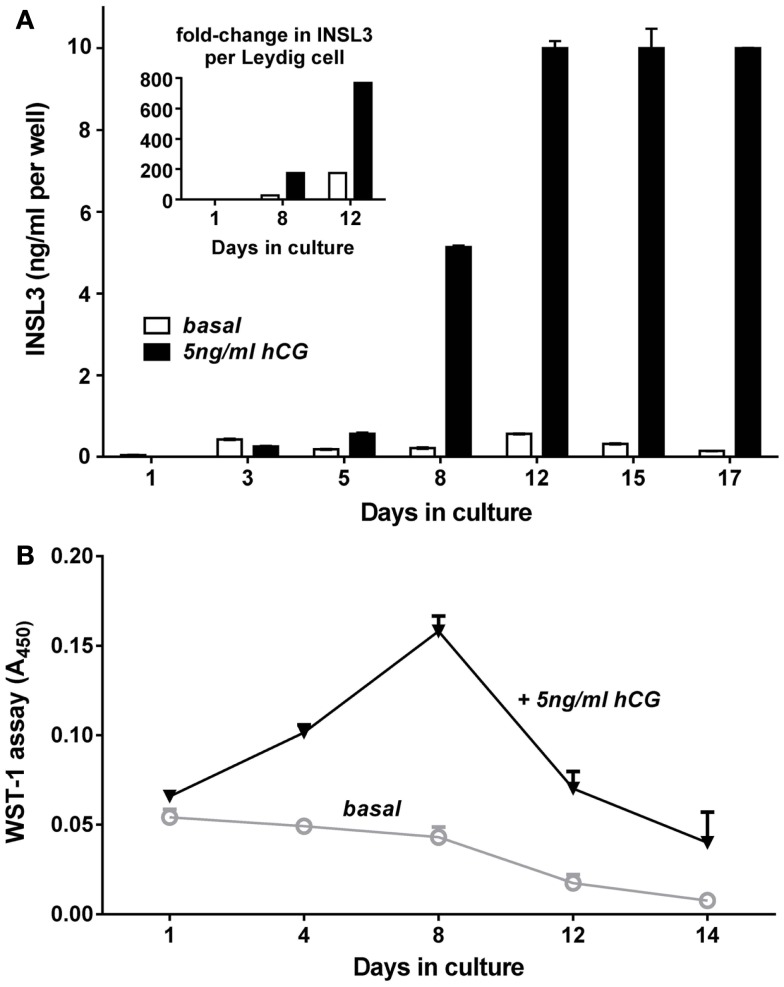
**Differentiation *in vitro* of post-natal day (PND) 10 rat Leydig cells in the absence (open bars) or presence (filled bars) of hCG**. **(A)** Cells were purified from the abdominal testes of PND10 male Sprague Dawley rats by mechanical dispersion followed by unit sedimentation, then cultured in serum-free medium at 400,000 cells per well of 12-well plates at 37°C. Medium was changed every 2–3 days, with aliquots collected exactly 48 h after the last medium change, for measurement of INSL3 using rat INSL3-specific TRFIA (10). **(B)** Cells prepared as above were seeded in parallel at 30,000 cells per well into 96-well plates and subjected to the WST-1 (4-[3-(4-Iodophenyl)-2-(4-nitro-phenyl)-2H-5-tetrazolio]-1,3-benzene disulfonate) assay to measure cell numbers, as described by the manufacturers (Roche Applied Science (Castle Hill, NSW, Australia). The inset in the upper panel indicates the fold-increase in INSL3 secretion calculated on a per cell basis for key times relative to basal expression on day 1, thus representing the differentiation of the individual Leydig cells, discrete from any effects on cell proliferation or cell death. This shows that while hCG has a marked effect on Leydig cell proliferation and/or survival, it is not essential for cell differentiation, though it does augment it. Animal experimentation was conducted under the terms of permit S-2010-102 of the Animal Ethics Committee, University of Adelaide.

Cell culture studies using either MA10 mouse tumor or primary adult rat Leydig cells show that INSL3 is largely a constitutive secretory product of Leydig cells, and is not acutely regulated by cAMP or LH (hCG) in the short-term (hours), unlike steroidogenic enzyme activity (10, 28). However, if Leydig cells are subjected to differentiation processes, by being allowed to dedifferentiate in culture, or by collecting cells from immature testes, then LH or hCG have a markedly stimulatory effect on INSL3 production (Figure 4), because the gonadotropins can induce both Leydig cell proliferation and augment differentiation, and hence increase INSL3 production, which is a chronic (days) differentiation-dependent process. It should be noted that *in vivo* INSL3 is a biomarker for late Leydig cell differentiation (6). In Figure 4, immature Leydig cells prepared from rats at post-natal day 10 initially express no INSL3, as *in vivo*. Without additional gonadotropin, there is already some differentiation and INSL3 expression. However, with regular addition of hCG (as a surrogate for LH), these immature Leydig cells first proliferate until about day 8 of culture, equivalent to about day 18 *in vivo*, and then start to differentiate, with some cells also dying in culture, as reflected by the WST-1 assay (Figure 4B). Once differentiated, the Leydig cells cease further multiplication.

The difference between LH-dependent testosterone production and LH-dependent INSL3 production is well illustrated by Figure 3, because here we see that during puberty in rats, INSL3 first overshoots in response to the massive bursts of LH production (without feedback regulation), unlike testosterone which is acutely regulated at the level of enzyme activity. As androgen feedback leads progressively to a stabilization of the HPG axis (after day 60 in the rat) at a more moderate LH level (the “thermostat” model), and a correspondingly reduced level of Leydig cell metabolism (differentiation status), then so are the circulating INSL3 levels reduced to reflect that stable Leydig cell functional capacity. This situation is made a little more complex because not only do Leydig cells differentiate under chronic LH influence, but also immature Leydig cells can proliferate in an LH-dependent manner. What INSL3 as a constitutive biomarker is measuring is the sum of both differentiation status (individual cell maturity) and cell number, which together is captured by the term Leydig cell “functional capacity.”

We have emphasized these important distinctions because the literature, particularly concerning INSL3 in hypothalamic hypogonadal men, is confusing [e.g., Ref. (29)]. Where such men are treated with hCG/LH for periods of less than a few days, there may be an acute increase in peripheral testosterone production, but there will be no change in circulating INSL3 (8). This is different where the hCG stimulus is chronic, for periods of weeks or months [e.g., Ref. (7, 29)]. The gonadotropin thereby induces the differentiation of the Leydig cells, thereby increasing their functional capacity, and concomitantly therefore increases also the levels of circulating INSL3. INSL3 is still being constitutively generated (in an acute sense) by those individual Leydig cells. Another example to illustrate this point is observed in uni-orchid men, who have one testis removed because of testicular cancer, but are otherwise healthy (9). Their Leydig cell functional capacity is obviously reduced compared to intact men, although those individual Leydig cells will be metabolically highly stimulated. Whereas, as expected, compensatory feedback to the HPG axis has caused a significant increase in LH and an almost normalization of testosterone levels, circulating INSL3 concentration remains significantly reduced (9), and in fact there is an inverse relationship between circulating LH and INSL3 concentrations (9). This is because where the number of Leydig cells is limiting, the number of Leydig cells will be simply reflected by the INSL3 concentration which will be independent of LH. However, the more Leydig cells present, the less LH is required to maintain normal testosterone levels according to the “thermostat” model, and hence the inverse relationship.

A further example to illustrate this point is seen in aging men. When men become old, their circulating testosterone declines at approximately 6% per decade after the age of 40. However, this is continually being compensated by increasing LH, reflecting the continued acute feedback regulation via the HPG axis. For INSL3, produced by the same Leydig cells, the reduction is much greater (ca. 12% per decade) because this acute feedback compensation does not occur (9).

This concept of Leydig cell functional capacity is otherwise best captured only by the ratio of T/LH (30, 31), which of course, unlike a constitutive marker such as INSL3, is subject to the technical variation of being able to reliably measure both T and LH (32, 33). Another feature which reflects this notion of INSL3 as a constitutive biomarker is its technical consistency. We have measured INSL3 in repeated blood samples from young men and have found <10% variation over periods of several months (Anand-Ivell and Ivell, unpublished). Not only is it a technically more robust parameter to measure, but because it is constitutively measuring Leydig cell functional capacity, and is thus not subject to acute feedback fluctuations, as are testosterone and LH, it represents a valuable biomarker, particularly to follow treatments to remediate hypogonadism (29), or to map the progression of puberty (34).

## Actions of INSL3 in the Testis

Besides the two known endocrine functions of INSL3, to induce the first transabdominal stage of testicular descent (35, 36), and to support bone metabolism and horn growth (37, 38), INSL3 appears to exert functions within the testis, thereby supplementing the conventional role of the HPG axis. The unique INSL3 receptor, RXFP2, has been identified at mRNA and at protein levels on both Leydig cells themselves (39), and also on germ cells within the seminiferous compartment (2, 39–41), but not on other testicular cell types.

Considering an autocrine/paracrine role within the interstitial compartment of the testis, it is important to recognize that under normal circumstances, the adult interstitial fluid will have constitutively high concentrations of INSL3 [in the rat, ca. 400 ng/ml; (10)], such that any surface RXFP2 receptors present are likely to be saturated and most likely desensitized [*K*_d_ <1 nM or <6 ng/ml; (1)]. Thus, any role for INSL3 in this compartment is likely to be relevant only in early puberty prior to the completion of Leydig cell differentiation, or similarly during early embryonic development for the fetal population of Leydig cells, or in equivalent disease states such as hypogonadism. In support of this, an interesting study by Pathirana and colleagues showed that INSL3 had a significant stimulatory effect upon Leydig cell steroidogenesis *in vitro*, but only where the cell density in culture was very low, and presumably endogenous INSL3 production was also low (42). Recent studies in the ovary using follicular theca cells, which are the female equivalent of Leydig cells, showed a similar stimulatory effect of INSL3 on theca cell steroidogenesis (18). This effect was absolutely dependent on RXFP2 expression, and could be reduced by transfecting cells with an RXFP2-specific siRNA (18). Thus, INSL3 appears to be part of a feed-forward mechanism buffering the production of steroids consequent upon LH stimulation, and may have most impact during the first spermatogenic wave before Leydig cells have fully differentiated.

RXFP2 is also expressed by male germ cells (39, 40). In particular, the INSL3 receptor is found modestly expressed by spermatocytes, and to a greater amount on post-meiotic germ cells (39). Experiments in rats show that ca. 20 ng/ml of INSL3 can reach the seminiferous compartment across the blood–testis barrier by mechanisms, which are still unclear (10). This is sufficient to have a modulatory role on male germ cells. Several pieces of evidence support a survival factor/anti-apoptotic role for INSL3 in regard to germ cells, thus effectively abetting the role of FSH acting via Sertoli cells (Figure 2). First, in rats, it was shown that INSL3 was able to reduce the amount of germ cell death by apoptosis following GnRH antagonist treatment (40). Second, injection of an INSL3 antagonist into rat testes led to a significant reduction in testis weight (43), presumably resulting from germ cell death. Third, in men subjected to a steroidal contraceptive regimen to suppress the HPG axis, it was found that men retained most residual spermatogenesis when their circulating INSL3 levels were highest (44).

Taken together, these results strongly suggest that INSL3 is acting as an intratesticular autocrine/paracrine system to buffer the conventional output from the male HPG axis, thereby reducing unnecessary fluctuations induced by extrinsic influences (e.g., stress) or excessive pulsatility within the HPG axis, and modulating both LH and FSH actions.

## INSL3 Synergy with Androgen Action

Insulin-like factor 3 has been described as a “neohormone” (45, 46), i.e., as a hormone which has evolved specifically to address functions uniquely linked to the mammalian phenotype and evolution. One of the most obvious of these roles is the promotion of testicular descent and a scrotal testis. But also its role to promote horn and bone growth in the male (38) is closely linked to male reproductive behavior, another typical neohormone parameter (46). Inspection of the mechanisms of INSL3 action both as an endocrine, as well as a paracrine/autocrine hormone, indicates that INSL3 is mostly synergizing directly or indirectly with gonadotropin-induced androgen action, for example in bone and horn growth, in maturation of the male tract in the embryo, and in supporting germ cell survival within the seminiferous tubules. Also in the female, where INSL3 is not a highly expressed circulating hormone, it acts in concert with LH, FSH, and androstenedione to promote follicle growth and steroid production (18, 47). The precise molecular details of this synergy are not yet clear, although there is a good evidence to suggest that androgen receptor activation is required for RXFP2 expression (17, 18), and that, at least in the action of INSL3 on the gubernaculum, signaling pathways are induced very similar to those induced by androgen action (19).

## INSL3 and Pathology

Since INSL3 is part of a synergistic network modulating gonadotropin action, highly specific effects of INSL3 alteration are not to be expected. A complete loss of function of INSL3 or its receptor in mice or humans is associated with osteopenia/osteoporosis (37) and cryptorchidism (35, 36). Whilst a loss of INSL3 in the ovary appears to be linked to a reduction in antral follicle growth and maturation (48), no such gross aberration is evident for the adult testis, even when the receptor knockout is specifically targeted to the testis to avoid any repercussions caused by cryptorchidism (49). However, this latter study did not look at those phases of development such as puberty or during insult situations when the buffering or modulatory effect of INSL3 is likely to be most evident. A reduced INSL3 production by fetal Leydig cells appears to be instrumental in some aspects of the testicular dysgenesis syndrome induced by intra-uterine exposure to endocrine disrupting agents, such as phthalates in rats [reviewed in Ref. (12)]. It is also useful as a monitor to measure effects on Leydig cell development and functional capacity [reviewed in Ref. (6)], being less subject to random fluctuation than androgens. A recent observation resulting from a study of 1200 normal men in Australia also needs to be pursued. It was shown in this study that even young healthy men showed substantial variation (>4-fold) in their circulating levels of INSL3, presumably reflecting a very varied Leydig cell functional capacity (9). Whilst the absolute levels of this hormone are probably still sufficient to support normal physiology, it poses the question as to the causes of such variation, and the long-term impacts, for example, in terms of supporting gonadotropin-induced androgen action later in life. Leydig cell numbers once established in puberty do not appear to change substantially during the remainder of life, there being very little evidence for Leydig cell loss or proliferation in the adult (50). Whilst in the human it has been reported that there is a loss of Leydig cells in old age (51), only recognizably mature cells were counted here, excluding cells which may have dedifferentiated. Longitudinal studies are needed here to explore these aspects further.

## Conclusion

Insulin-like factor 3 is an important new downstream effector of the HPG axis, which in the male, unlike androgens, does not appear to be subject to acute fluctuation, but through positive feed-forward mechanisms, rather acts to buffer the stimulus of LH (directly via Leydig cells) and of FSH (indirectly via Sertoli cells) on both steroidogenesis as well as germ cell production, respectively (Figure 2). Moreover, as a constitutive measure of Leydig cell functional capacity, it also acts as a kind of “memory” for historical insults which may during development, and possibly also in later life, have impacted on the final capacity of the testes to produce androgens.

## Author Contributions

Richard Ivell was responsible for the drafting of the manuscript. Ravinder Anand-Ivell was responsible for the overall conception of the manuscript and contributed substantially to the drafting, as well as carrying out a number of the experiments reported. Kee Heng carried out several experiments reported in this manuscript as part of her PhD thesis at the University of Adelaide. All authors have read and agree to the finally submitted text.

## Conflict of Interest Statement

This work was funded in part by the National Health and Medical Research Council of Australia (project grant no. 1009243). The funding agency had no influence over the content or results presented in this article.
